# [Retracted] RNA interference‑mediated USP22 gene silencing promotes human brain glioma apoptosis and induces cell cycle arrest

**DOI:** 10.3892/ol.2023.13756

**Published:** 2023-03-13

**Authors:** Zhao Hui Li, Yin Yu, Chao Du, Hong Fu, Jian Wang, Yu Tian

Oncol Lett 5: 1290–1294, 2013; DOI: 10.3892/ol.2013.1188

Following the publication of this paper, it was drawn to the Editor's attention by a concerned reader that certain of the cell cycle plots shown in Fig. 5A, and western blotting data in Fig. 6A, on p. 1293 were strikingly similar to data appearing in different form in other articles by different authors at different research institutes. Owing to the fact that the contentious data in the above article had already been published, or were already under consideration for publication, elsewhere prior to its submission to *Oncology Letters*, the Editor has decided that this paper should be retracted from the Journal. After having been in contact with the authors, they did not agree with the decision to retract the paper. The Editor apologizes to the readership for any inconvenience caused.

